# Does stereotactic body radiation improve outcomes compared to conventional radiation for liver cancer patients?

**DOI:** 10.1016/j.ctro.2022.04.002

**Published:** 2022-04-11

**Authors:** Michael Lock, Ronald Chow, Aruni Jayatilaka, Meghan Plotnick, Robert Stephens, Timothy Nguyen, Barbara Fisher, Eugene Wong, Steward Gaede

**Affiliations:** a Schulich School of Medicine & Dentistry, University of Western Ontario, London, ON, Canada; b Temerty Faculty of Medicine, University of Toronto, Toronto, ON, Canada; c Faculty of Medicine, Dalhousie University, Halifax, NS, Canada

**Keywords:** Liver cancer, Radiotherapy, Fractionation, Hypofractionation

## Abstract

**Introduction:**

Over the past decade, there has been a paradigm shift to higher doses per fraction of liver stereotactic body radiotherapy (SBRT). However, this shift may not be due to evidence alone, but rather result of convenience and remuneration. This prospective cohort study aims to compare outcomes of patients who received moderately hypofractionated and hypofractionated radiotherapy treatments for liver tumours.

**Methods:**

Patients treated for liver cancer with radiotherapy between 2004 and 2020 were prospectively entered in this study. Patients were stratified into two groups: hypofractionation group of patients receiving an average of 5 fractions, and moderate hypofractionation group of patients receiving an average of 17 fractions. Other components of precision radiotherapy such as image guidance were the same between groups. The primary outcome was 2-year overall survival. The secondary outcomes were (1) change in toxicity as assessed by the Radiation Therapy Oncology Group (RTOG) toxicity criteria from baseline to 3 months, and from baseline to 6 months; and (2) change in Child Pugh score from baseline to 3 months. Type I error was prespecified at 0.05.

**Results:**

397 patients were included. A larger proportion of patients on hypofractionated regimens were alive at the 2-year time point, relative to those who received moderately hypofractionated regimens (42% vs 27% *p* = 0.010); no difference was noted at the 1-year time point. Mean toxicity change in RTOG symptoms from baseline to 3 and 6 months, and in Child Pugh score from baseline to 3 months, were not statistically different between the two groups.

**Conclusion:**

When compared to conventional radiation fractionation, liver SBRT tends to be associated with a significant overall survival benefit, justifying a randomized trial to confirm. The concern that the trend to higher dose per fraction may result in increased toxicity, specifically in the treatment of high-risk liver patients, appears to be unfounded. The hypofractionated component of SBRT may be the critical dosimetric factor impacting on survival.

## Highlights

• There has been a paradigm shift to SBRT without evidence that these high-dose ultra-low fractions result in improved outcomes.
• This prospective cohort compares the survival of patients treated with conventional versus SBRT treatments for liver tumours.
• The SBRT group received an average of 5 fractions, and the conventional group received an average of 17 fractions.
• 397 patients were included. Overall survival was higher for SBRT patients at the 2-year time point (42% vs 27% *p* = 0.01).

## Introduction

Before 2010, the use of radiotherapy in the treatment of liver tumours was limited due to safety concerns with respect to radiation-induced liver disease (RILD). RILD is a syndrome consisting of anicteric ascites and hepatomegaly, and elevation of alkaline phosphatase relative to other liver transaminases. The downstream effect was an increased risk of progression to liver failure which may be life threatening [1]. Initial investigation of liver radiation involved increasing radiotherapy fraction number with the goal of increasing the dose to ablative levels to replace surgery, the standard of care. Similar dose escalation programs for other disease sites, such as prostate cancer, had shown improved clinical outcomes with dose escalation achieved using increased number of fractionations. However, hyperfractionation becomes less practical as doses, considered ablative, are reached; common fractionations to achieve ablative doses commonly exceeded 2 months and still did not result in sufficient clinical control [2]. Positive sequential phase I and II trials quickly led to widespread adoption of hypofractionation as integral to the new concept of stereotactic body radiotherapy (SBRT) [3].

Over the past decade, significant advances in radiotherapy delivery, particularly intensity modulated radiation treatment planning, image guidance and a better understanding of partial volume irradiation radiobiology have allowed for safe delivery of radiotherapy for patients with liver tumours [4]. As a result, treatment for liver tumours has evolved to include radiotherapy as a valuable option for patients with few alternatives [5], [6]. SBRT has essential components of (1) specific quality assurance (QA) measures, especially image guidance for motion (2) a dose at least equivalent to conventional radical doses, and (3) use of up to a maximum of 10 fractions and usually less than 6 fractions [7]. This definition has widespread agreement including the American Association of Physics in Medicine (AAPM) Task Group 101 [8]; the American Society for Therapeutic Radiology and Oncology and the American College of Radiology (ASTRO and ACR) [9]; the Canadian Association of Radiation Oncology—Stereotactic Body Radiotherapy (CARO-SBRT) [10] and the National Radiotherapy Implementation Group of the UK [11]. This paradigm shift to higher doses per fraction has been a dominant treatment concept over the past few years especially with the availability of new technology [14]. But the switch to hypofractionation may have been driven more by factors such as convenience and remuneration. Indeed, some noteworthy early trials suggested that hypofractionation, particularly in subgroups such as Child Pugh B patients, provided no additional benefit [12], [13]. Therefore, as part of one of the earliest image-guided and radiobiologically-guided liver radiation programs, we compared ‘moderate hypofractionation’ (conventional) to hypofractionation (SBRT) in a prospective study.

To date, ‘moderate hypofractionation’ (usually defined as greater than 10 fractions) versus SBRT hypofractionation regimens (defined as less than or equal to 6 fractions) have not been well-studied. The aim of this study is to report on a cohort study, comparing outcomes of patients who received moderately hypofractionated and hypofractionated radiotherapy treatments for liver tumours. All other essential components of SBRT were similar in each group.

## Methods

Patients treated for primary or metastatic liver cancer with radiotherapy between 2004 and 2020 were prospectively entered in this study. All data and time-points were selected prospectively and approved by the institutional ethics committee (Western University REB # R-09–506). However, the current analysis was conducted retrospectively using this database. The study was a sequential cohort design and non-randomized. Patients were included if they consented, had a liver cancer diagnosis, had a Zubrod performance status of 0–2, and had a Child-Pugh score of ≤ B7 within 14 days of study enrollment. Patients who received 1 fraction with palliative intent were excluded. Extrahepatic disease was allowed, as were oligoprogressive lesions. All patients underwent treatment and simulation according to a standardized protocol. A triphasic computed tomography (CT) simulation was performed with four-dimensional computed tomography (4DCT) in the supine position in a body immobilizer. Fiducial markers were not standardly used. MRI fusion was only performed in cases where CT was known to have poor visualization or patients had contrast allergies. A subset average respiratory-gated treatment technique was used with daily image guidance using the Real-Time Position Management (RPM) System (Varian Medical Systems, Palo Alto, CA). The dose was escalated using radiobiological guidance similar to the RTOG 1112 and Princess Margaret Hospital protocol, where tumor control probability (TCP) was escalated to a maximum normal tissue complication probability (NTCP) of 5% [15].

Clinical profiles of patients were recorded at baseline prior to radiotherapy treatment, as well as any previous treatment they received. Patients were stratified into two groups: hypofractionation group of patients receiving 2–6 fractions, and ‘moderate hypofractionation’ group of patients receiving greater than 6 fractions. Patients were treated in accordance to the standards of practice at the time, and therefore over the past two decades, there are patients who were treated with hypofractionation or moderate hypofraction. The prospectively determined follow up protocol included imaging and labs at one-month post treatment, and then every three months.

The primary outcome was 1-year and 2-year overall survival. The secondary outcomes were (1) change in toxicity as assessed by the Radiation Therapy Oncology Group (RTOG) toxicity criteria from baseline to 3 months, and from baseline to 6 months; and (2) change in Child Pugh score from baseline to 3 months.

Parametric tests were used to compare clinical profiles (means and proportions) between the two groups, as well as the outcomes. Type I error was prespecified at 0.05. All analyses were conducted using SAS 9.4. Analysis of this dataset was approved by the Office of Research Ethics at the Western University.

## Results

### Patient and treatment characteristics

A total of 397 patients were included in our analysis, accrued between 2004 and 2020. Mean age was 65 years, and 37% were male. Patient demographics for the entire cohort, and by fractionation group, are presented in Table 1. A significantly larger proportion of patients receiving hypofractionated treatment had cirrhosis (*p* = 0.042). Patients who received moderately hypofractionated regimens received an average of 17.2 fractions (range: 7–30), whereas those who received hypofractionated regimens received an average of 5.3 fractions (*p* less than 0.001) as our SBRT treatment was initially 6 fractions for many years. The median physical dose was 52.0 Gy for the moderately hypofractionated group, and 37.5 Gy for the hypofractionated group. The median equivalent dose in 2 Gy per fraction (EQD2) was 60.0 for the moderately hypofractionated group and 57.0 for the hypofractionated group. The alpha/beta used was 10 Gy. The median biological effective dose was 72.0 and 68.3, respectively. The median tumour size was 6.1 cm and 6.2 cm for the moderately hypofractionated and hypofractionated group, respectively. The Child Pugh score at baseline was a median of 5 for both groups. Over half of radiated patients having received prior chemotherapy. Of the hypofractionated group, 43% had extrahepatic disease, compared to 52% in the moderately hypofractionated group. The 1-year local control rate was 78% and 84% for patients receiving moderately hypofractionated and hypofractionated regimens, respectively; the 2-year local control rates were 78% and 82%, respectively. There was no significant difference was noted between groups, with respect to local control rates.

**Table 1:** Patient and Treatment Demographics.

	≤ 6 Fractions (n = 305)	> 6 Fractions (n = 92)	Total (n = 397)
Age (Years)	65.3 ± 13.2	64.3 ± 14.2	65.1 ± 13.4
Male	112 (37%)	36 (39%)	148 (37%)
Number of Fractions*	5.3 ± 0.6	17.2 ± 9.4	8.1 ± 6.8
Primary Cancer Diagnosis			
Hepatocellular	98 (32%)	25 (27%)	123 (31%)
Colorectal	77 (25%)	29 (32%)	106 (27%)
Cholangiocarcinoma	27 (9%)	20 (22%)	47 (12%)
Breast	23 (8%)	4 (4%)	27 (7%)
Lung	16 (5%)	4 (4%)	20 (5%)
Other	64 (21%)	10 (11%)	74 (19%)
Largest Tumor Size (cm)	6.1 ± 4.1	6.2 ± 3.8	6.1 ± 4.0
Ascites	44 (14%)	10 (11%)	54 (14%)
Hepatitis	53 (17%)	12 (13%)	65 (16%)
Cirrhosis*	72 (24%)	13 (14%)	85 (21%)
Extrahepatic Disease	132 (43%)	47 (52%)	179 (45%)
Previous Treatment			
Chemotherapy	156 (51%)	54 (59%)	210 (53%)
Resection	53 (17%)	14 (15%)	67 (17%)
Abdominal Radiotherapy	38 (13%)	3 (3%)	41 (10%)
Radiofrequency Ablation	24 (8%)	3 (3%)	27 (7%)
Median Physical Radiation Dose	37.5 Gy	52 Gy	42 Gy

*Note: statistically significant difference (*p* < 0.05) between two fractionation regimens

### Survival

A larger proportion of patients on hypofractionated regimens were alive at the 2-year time point, relative to those who received moderately hypofractionated regimens (42% vs 27% *p* = 0.010); no difference was noted at the 1-year time point (Table 2).

**Table 2:** Outcomes.

	≤ 6 Fractions (n = 305)	> 6 Fractions (n = 92)	Total (n = 397)
Overall Survival			
1 Year	161 (53%)	40 (43%)	201 (51%)
2 Year*	128 (42%)	25 (27%)	153 (39%)
Toxicity Change			
0-3 Months	0.35 ± 1.09 (n = 183)	0.19 ± 0.90 (n = 74)	0.30 ± 1.03
0-6 Months	0.32 ± 1.23 (n = 167)	0.33 ± 1.09 (n = 57)	0.32 ± 1.20
Child Pugh Score Change			
0-3 Months	-1.65 ± 2.24 (n = 158)	-1.11 ± 2.57 (n = 36)	-1.55 ± 2.31

*Note: statistically significant difference (*p* < 0.05) between two fractionation regimens

### Toxicities

Mean toxicity RTOG symptom change score (1.07 for hypofractionation, and 0.91 for moderately hypofractionated patients) was not different between the two groups, between baseline, and the time points at 3 and 6 months. Child Pugh score at baseline was noticeably higher in patients receiving hypofractionated treatments (5.87 ± 1.21 vs 5.26 ± 1.25; *p* less than 0.001). However, the two treatment groups experienced similar change in toxicity and Child Pugh Score (Table 2).

## Discussion

SBRT is increasingly being used for the management of many cancers especially those where previous attempts at radiotherapy control have been suboptimal such as hepatobiliary and pancreatic cancers. The hypofractionation component of SBRT has been especially appealing during the COVID-19 pandemic [16], [17] One of the most compelling reasons for this rapid pivot in management may be due to the reduced burden on patients’ time and resources, and reduced cost to the healthcare system [18]. However, there is concern that the move to SBRT may be premature as high dose per fraction may lead to an increased toxicity risk without clear evidence that this type of hypofractionation would improve outcome. Indeed, recent studies have confirmed findings from early SBRT studies where no difference was found between SBRT and conventional radiation [13], [19]. We sought to compare SBRT to conventional radiation focussing on the impact of the hypofractionation component of SBRT with all other components of SBRT being similar.

This study reports that patients receiving hypofractionated and moderately hypofractionated regimens experience similar toxicity and confirms the relative safety of radiation for liver lesions with comparable results seen in the literature [20], [21], [22]. For the principle end-point, a significantly improved overall survival at the 2-year time point was found, despite radiobiological dose equivalence between cohorts. Furthermore, as the dose per fraction in SBRT studies has increased, critics of SBRT have raised the concern that the higher dose per fractions regimens would result in an increased risk of toxicity even when the dose regimens had calculated dose equivalence as determined by biologically equivalent doses (BED) and EQD2 measures. However, this study demonstrates that conventional and hypofractionated regimens are similarly safe suggesting that we can safely move to SBRT with similar clinical safety and improved survival outcomes.

Another concern with SBRT, is that certain subgroups may be at higher risk of toxicity with high dose per fraction regimens. Hepatic irradiation patients are known to be quite heterogeneous and there may be subgroups that may benefit from higher fractionation for toxicity that this sample size may not have the power to identify. For example, patients with larger tumors and higher Child Pugh scores may be offered a higher fractionated regimen to decrease the dose per fraction in some centres. Indeed, a landmark trial published by Cardenes *et al* has recommended essentially this approach of increasing the number of fraction regimen for the subgroup Child-Pugh ≥ B7 patients where a RILD toxicities were identified. After this change, no further RILD cases were noted in the Indiana formal dose escalation series [23]. However, this trial also instituted dose changes for this subgroup and more restrictive organ-at-risk constraints which may have been the real reason for the improved toxicity profile. Active trials such as ABC-07 for locally advanced bile duct cancer specifically recommend a 15 fraction regimen over a 5 fraction regimen for the subgroup of patients with larger tumours [24]. We assessed whether moderate or SBRT hypofractionation for larger tumours resulted in greater toxicity or improved survival, but no difference could be detected. As for other subgroups, we assessed those with poor liver function, heavily pre-treated with interventions (such as chemotherapy), and older age group, but could not identify a particular subgroup that had a clinically important statistical signal suggesting they benefit from higher number of fractions while keeping the dose equivalent in terms of toxicity or, conversely, be harmed in terms of survival or local control.

Primary and secondary cancers were assessed as one statistical group for impact of hypofractionation versus conventional treatment. No difference could be identified and the primary and secondary cancers were merged to increase statistical power. The lack of impact of disease site suggests that, at least in terms of fractionation, radiation is agnostic to the type of cancer. There is data that suggests a difference in dose needed for ablation of primary versus secondary hepatic lesions. For example, data from Lausch et al. [12] demonstrated that an EQD2 of 53 Gy was required for hepatocellular carcinoma while 84 Gy was required for metastatic lesions. This highlights the sensitivity of hepatocellular carcinoma and relative radioresistance of the often heavily pretreated nature of patients with metastases. However, unlike the early work by Lausch et al. [12], we did not see a difference when grouped by moderate hypofractionation versus hypofractionated treatment. A standard dose regimen for either primary or metastatic liver disease has not been established in the literature or based on this data, so institutions can offer the same dose fractionation that is known to be safe to primary or secondary cancers to improve uniformity of liver cancer radiation delivery. Based on this study, this should be less than 6 fractions.

Strengths of this trial include the prospective data collection where prespecified data are collected at uniform time points in a standardized manner for both cohorts. This includes imaging, liver enzymes and liver function at 6 weeks post treatment and then every 3 months that allowed for a more uniform data collection between groups. Another strength of the study was that the sample size was sufficiently powered to detect a clinically important difference. Based on the rule of 3, the chance that there is a toxicity difference is less than 1 in 100–150 [25]. As this is one of the largest single center North American databases, this provides one of the best estimates of harm for patients and provides confidence in our shift to the current definition of SBRT where fractionations are 5 or less.

However, this study has limitations worth noting. These results are drawn from a single centre sequential cohort study and generalizability to other patients or institutions should be confirmed. As this was not randomized, important differences in patient characteristics may not have been equally distributed between groups. There may be a time series bias and a “learning curve”, as those patients in the moderate hypofractionation cohort were treated when the high precision program was starting. This prospective study was started in 2004 when much of our current understanding of SBRT was still being established. This includes factors such as ablative doses, fractionation, SBRT normal tissue constraints, lesion size, number of lesions, and the importance of tools and constraints that could estimate the risk of toxicity such as the Child Pugh Score. As experience increased in the group, we opened up the eligibility to higher risk patients, higher risk disease sites (e.g., percent of patients with cholangiocarcinoma increased to 22% compared to 9% in the initial cohort) and higher risk anatomy (e.g., proximity to luminal structures) as the data on the safety of SBRT was accumulating in the department and in the world literature. As well, full standardized quality-of-life (QOL) data was not available, especially for the moderately hypofractionated group; there may be a difference between fractionation groups that could not be determined in this study and may highlight the importance of QOL data collection for other trials investigating fractionation in hepatic disease.

In conclusion, in this non-randomized comparison of SBRT versus conventional fractionation with similar EQD2, this study was able to demonstrate that the concern that the trend of higher dose per fraction may result in an increased risk of toxicity appears to be unfounded. Hypofractionated liver SBRT, defined as 5 or less fractions, seems to result in a significant overall survival benefit, which provides support for the current trends in SBRT as a whole and in liver radiotherapy in particular.

## Declaration of Competing Interest

The authors declare that they have no known competing financial interests or personal relationships that could have appeared to influence the work reported in this paper.
